# Polymorphisms of Dopamine Receptor Genes and Risk of L-Dopa–Induced Dyskinesia in Parkinson’s Disease

**DOI:** 10.3390/ijms18020242

**Published:** 2017-01-24

**Authors:** Cristoforo Comi, Marco Ferrari, Franca Marino, Luca Magistrelli, Roberto Cantello, Giulio Riboldazzi, Maria Laura Ester Bianchi, Giorgio Bono, Marco Cosentino

**Affiliations:** 1 Movement Disorders Centre, Neurology Unit, Department of Translational Medicine, University of Piemonte Orientale, 28100 Novara, Italy; magis.luca@gmail.com (L.M.); cantello@med.uniupo.it (R.C.); 2 Center of Research in Medical Pharmacology, University of Insubria, 21100 Varese, Italy; marco.ferrari@uninsubria.it (M.F.); franca.marino@uninsubria.it (F.M.); marco.cosentino@uninsubria.it (M.C.); 3 Departments of Biotechnology and Life Science, University of Insubria, 21100 Varese, Italy; g.riboldazzi@fastwebnet.it (G.R.); mlebianchi@hotmail.com (M.L.E.B.); giorgio.bono@uninsubria.it (G.B.)

**Keywords:** Parkinson’s disease, SNPs, personalized medicine, disease progression, motor complications

## Abstract

L-dopa–induced dyskinesia (LID) is a frequent motor complication of Parkinson’s disease (PD), associated with a negative prognosis. Previous studies showed an association between dopamine receptor (DR) gene (*DR*) variants and LID, the results of which have not been confirmed. The present study is aimed to determine whether genetic differences of *DR* are associated with LID in a small but well-characterized cohort of PD patients. To this end we enrolled 100 PD subjects, 50 with and 50 without LID, matched for age, gender, disease duration and dopaminergic medication in a case-control study. We conducted polymerase chain reaction for single nucleotide polymorphisms (SNP) in both D1-like (*DRD1*A48G; *DRD1*C62T and *DRD5*T798C) and D2-like *DR* (*DRD2*G2137A, *DRD2*C957T, *DRD3*G25A, *DRD3*G712C, *DRD4*C616G and *DRD4*nR VNTR 48bp) analyzed genomic DNA. Our results showed that PD patients carrying allele A at *DRD3*G3127A had an increased risk of LID (OR 4.9; 95% CI 1.7–13.9; *p* = 0.004). The present findings may provide valuable information for personalizing pharmacological therapy in PD patients.

## 1. Introduction

Levodopa-induced dyskinesia (LID) is a disabling motor complication of long-term levodopa therapy in Parkinson’s disease (PD) [1,2]. The risk factors of LID include young age at PD onset, severe degeneration of nigrostriatal neurons, longer exposure and higher total daily dose of levodopa [2]. The pathophysiology of LID is quite complex and not fully understood. Nonetheless, strong evidence supports the contribution of dopamine as a major player in LID development [3,4,5].

The existence of profound inter-individual heterogeneity suggests that genetic predisposition may be a relevant determinant of LID [2,6]. Several variants of *DR* genes have been detected, and their role has been characterized in Alzheimer’s disease, schizophrenia, bipolar disorder, and addiction [7,8,9]. Work from our group showed that the TT genotype at *DRD1* rs686 may predispose PD patients to developing visual hallucinations (VHs) while subjects with GG at *DRD1* rs4532 display a shorter time to VHs [10]. A few studies explored the possible influence of *DR* variants on LID development in PD, but the reported results were not subsequently confirmed. One study showed that PD patients carrying the rs6280 single nucleotide polymorphism (SNP) at *DRD3* have earlier onset of peak dose dyskinesia [11]. An additional, more recent report suggested a higher risk of LID in patients carrying the TTCTA haplotype at the *DRD2*/*ANKK1* region [12]. Finally, Kaplan and colleagues did not find a significant correlation between SNPs at *DR*D2 and LID [13].

Due to the lack of confirmed results on the role of polymorphic *DR* variants in LID development, the present pilot study was designed to further investigate *DR* genetics in a small but accurately characterized cohort of Italian PD patients. To this end, we selected a panel of *DR* variants, giving priority to the most frequent and functionally characterized ones, and compared the frequency of all variants in two matched subgroups of PD patients with and without LID.

## 2. Results

### Dopamine Receptor (DR) Genotypes

There were no significant differences in demographic and clinical characteristics between patients with and without LID (Table 1). All *DR* alleles were in Hardy-Weinberg equilibrium (data not shown), and we did not find any linkage disequilibrium between considered SNPs. The frequencies of *DR* genotypes in patients with and without LID are shown in Table 2. Using Fisher’s exact test (recessive model), we found that the risk of LID was, on average, 4.9 (95% CI 1.7–13.9) times higher in subjects carrying the A allele in the rs6280 (25G>A) of the *DRD3*, *p* = 0.004. The same results were obtained using dominant and codominant models (data not shown). This significant association was confirmed by a two-way ANOVA test (*p* = 0.002). Moreover, we found a trend for an association between the A allele in rs1800497 (2137G>A) and LID, *p* = 0.010; however, this association was not statistically significant after the Bonferroni correction. No other SNP studied was associated with LID (Table 3).

**Figure 1:**
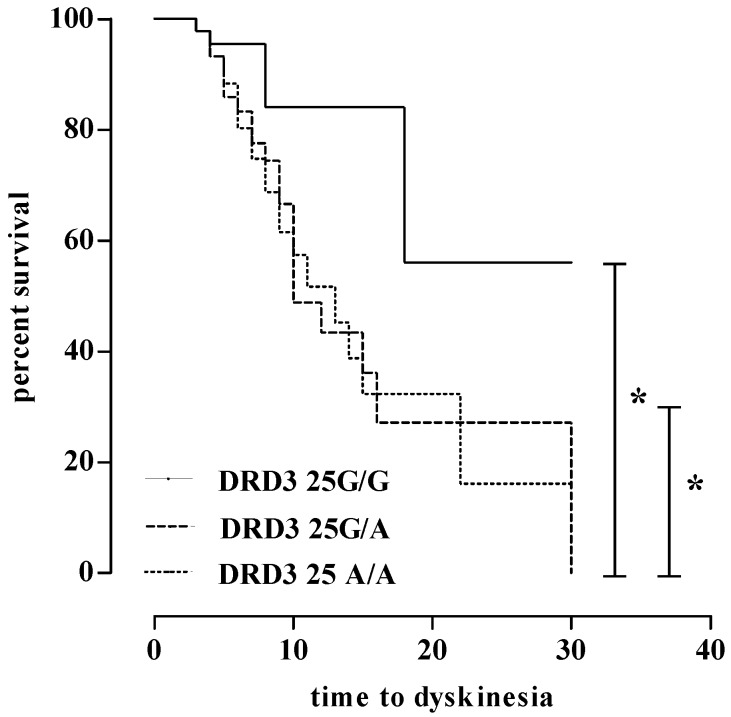
Correlations between rs6280 (*DRD* G25A) and time to dyskinesia. * = *p* < 0.005.

In Kaplan–Meier analysis, patients with *DRD3* rs6280 AA and AG had significantly shorter times to LID when compared with patients with the GG genotype (median 10 and 13, respectively, vs. 18 years; log rank *p* = 0.005, see Figure 1). No other SNP studied was associated with the timing of LID onset.

## 3. Discussion

This study provides the first evidence in an Italian cohort of PD patients that *DR* variability may predispose a person to LID. In fact, we found that the *DRD3* G25A variant at rs6280 was independently associated with LID development after adjusting for gender, age at PD onset, H&Y stage, and duration of levodopa treatment. Furthermore, this variant was also associated with an earlier development of LID. Our results confirm a previous observation showing an association between the G25A allele at rs6280 *DRD3* and an earlier onset of peak dose dyskinesia in Korean PD patients [11]. In addition, we also replicated the negative findings of Kaplan and colleagues regarding the possible correlations between SNPs at *DRD2* and LID [13]. As regards the other *DR* SNPs analyzed in our study, there are no previously published data on PD patients. Our findings are negative, but we cannot exclude that one or more of such *DR* variations might show some relevance in other PD populations.

The precise mechanism through which the D3 receptor predisposes one to LID is open to discussion. Data on monkey models of PD showed that D3 expression was more abundant in animals with LID than in those without LID [27]. Furthermore, the *DRD3* SNP rs6280 was shown to provide a higher binding affinity to dopamine [21] but also a higher susceptibility to tardive dyskinesia in patients with psychosis [28,29]. Dopamine receptor hypersensitivity may indeed be a possible mechanism involved in LID development [30], and the higher frequency of the *DRD3* G25A genotype in PD patients with LID may be explained by a role of this variant in the sensitization process of the basal ganglia circuitry.

Along with dopamine, a number of reports indicate that other signaling pathways may be of relevance in the context of LID predisposition. Genetics of adenosine, serotonin, glutamic acid and endocannabinoid receptors have been investigated [31], and the exclusive focus of our study on dopamine receptor genetics may be seen as a limitation. Nonetheless, our purpose was to shed light onto a series of previous hypotheses implying the genetics of dopamine pathways in LID predisposition. Such hypotheses were intriguing, but had never been confirmed before.

The possibility that clinical differences in the two patient groups may have influenced the findings of our study was carefully evaluated. Therefore, we performed a strict sample matching: for each patient with LID we selected a paired patient with the same features, i.e., gender, age at onset, disease duration, and therapy. Such a rigorous design had the downside of markedly restricting the sample size. Indeed, the main limitation of our study lies in the relatively small number of enrolled patients, which nonetheless reached the minimum estimated size to assess [10,32]. A further limitation is the candidate gene approach. Indeed, a more extensive analysis with direct sequencing of the five *DRs* might add relevant information to uninvestigated variations possibly playing a role in the genetics of LID.

## 4. Patients and Methods

### 4.1. Patients

We enrolled 100 consecutive patients with idiopathic PD: 50 patients who had experienced LID during their disease course, and 50 who had never complained of LID. Patients were all Caucasian Italian and were matched for age, gender, disease duration and treatment. The main features of the study population are shown in Table 1. Patients were enrolled at the Movement Disorders Centers of the University of Piemonte Orientale, Novara, and University of Insubria, Varese, Italy according to the following inclusion criteria: (1) clinical diagnosis of PD according to the UK Parkinson’s Disease Society Brain Bank criteria [33]; (2) age at onset >40 years; (3) active and longitudinal follow-up >4 years; (4) treatment with levodopa; (5) reliable data concerning time of levodopa treatment initiation and time of LID presentation. The study was approved by the local Ethics Committee (Novara, protocol number 9606) and patients were enrolled after having read and signed an informed consent form [10].

Outcome measures were obtained retrospectively from clinical records, in the context of a larger collaborative initiative aimed at identifying the genetic determinants of PD progression [10]. A review of available data in routine clinical records of each center was performed and agreement was found on the following evaluations: detailed collection of patients’ history, complete neurological examination, Unified Parkinson’s Disease Rating Scale (UPDRS) score, Hoehn and Yahr (H&Y) stage, presence and time to development of LID [10].

All patients had undergone a longitudinal follow-up with assessments every three to six months performed by a neurologist expert in movement disorders. UPDRS score, H&Y stage, total L-dopa daily dose equivalent (LED), were recorded at the time of the event in patients with LID and at an equal time point from onset of PD in each paired patient without LID. LED was calculated according to Tomlinson et al. [34].

### 4.2. Genotyping

Samples of 3 mL venous blood were collected from each patient and genomic DNA was obtained using a standard DNA extraction protocol (Qiagen Inc., Hilden, Germany). The following *DR* variants: rs4532 (−48A>G and rs686 (62C>T) in *DR*D1; rs1800497 (2137G>A) and rs6277 (957C>T) in *DR*D2; rs6280 (25G>A) and rs1800828 (−712G>C) in *DR*D3; rs747302 (−616C>G), and 7 48-base pair VNTR in *DR*D4; and rs6283 (978T>C) in *DR*D5 were analyzed by real time PCR using a GeneAmp 9700 PCR System (ABI, Foster City, CA, USA) and pre-designed genotyping assays (ABI). The *DRD4* 7 48-base pair variable number tandem repeat (VNTR) was examined using a previously published method [35]. An example of PCR curve for each SNP is included in Figure S1.

### 4.3. Statistics

Genotype frequencies were analysed by the two-way ANOVA test, χ^2^-test for trend or by the Fisher’s exact test, as appropriate, and the odds ratio (OR) with 95% confidence interval (CI) was calculated using dominant, codominant and recessive model. Kaplan–Meier (KM) plot was used for correlations between patient genotype and time to LID. Curves were compared using Log-rank (Mantel–Cox) test [10]. Bonferroni correction was applied when multiple comparisons were performed. A *p*-value ≤ 0.005 was considered statistically significant [10]. Presence of linkage disequilibrium was investigated using Haploview software [10].

## 5. Conclusions

In conclusion, our data provide a solid base towards the personalization of PD treatment since they may help in identifying fragile PD patients who would benefit from a less aggressive dopaminergic treatment.

## Figures and Tables

**Table 1:** Demographic and clinical features of study population.

Feature	No Dyskinesia	Dyskinesia	*p*
Number of subjects	50	50	
Gender, male/female	28/22	28/22	na
Age at onset, mean ± SD	65.1 ± 5.6	63.3 ± 9.8	ns
Disease duration (years) mean ± SD	10.8 ± 4.2	12.1 ± 5.2	ns
Dyskinesia onset (years) mean ± SD	na	7.6 ± 4.2	na
UPDRS III, mean ± SD *			
ON	24 ± 10	23 ± 9	ns
OFF	29 ± 13	28 ± 12	ns
Hoehn and Yahr, median (range) *	3 (1–4)	3 (1–4)	na
L-dopa treatment duration (years) mean ± SD	8.9 ± 3.4	9.6 ± 3.3	ns
Medication dose LED (mg/day), mean ± SD *	612.6 ± 242.6	741 ± 279.6	ns

* These variables were collected at time of event in patients with dyskinesia and at an equal time point from onset in each paired patient without dyskinesia; ns: not significant; na: not applicable; SD: standard deviation.

**Table 2:** Dopamine Receptor (*DR*) Frequency in Parkinson’s disease (PD) patients with and without dyskinesia.

Gene	SNP	Genotype	No Dyskinesia Dyskinesia Dyskinesia	Dyskinesia	P (a)	P (b)	OR (95% CI)
*DRD1*	rs4532	A/A	11 (22%)	11 (22%)	ns	ns	ns
		A/G	21 (42%)	20 (40%)			
		G/G	18 (36%)	19 (38%)			
	rs686	C/C	17 (34%)	15 (30%)	ns	ns	ns
		C/T	25 (50%)	25 (50%)			
		T/T	8 (16%)	10 (20%)			
DRD5	rs6283	T/T	33 (66%)	35 (70%)	ns	ns	ns
		T/C	13 (26%)	15 (30%)			
		C/C	4 (8%)	0 (0%)			
DRD2	rs1800497	G/G	36 (72%)	22 (44%)	ns	ns	ns
		G/A	11 (22%)	25 (50%)			
		A/A	3 (6%)	3 (6%)			
	rs6277	C/C	21 (42%)	11 (22%)	ns	ns	ns
		C/T	20 (40%)	28 (56%)			
		T/T	9 (18%)	11 (22%)			
DRD3	rs6280	G/G	26 (52%)	9 (18%)	0.0001	0.0001	4.9 (2.0–12.2)
		G/A	18 (36%)	21 (42%)			
		A/A	6 (12%)	20 (40%)			
	rs1800828	G/G	38 (76%)	39 (78%)	ns	ns	ns
		G/C	8 (16%)	11 (224%)			
		C/C	4 (8%)	0 (0%)			
DRD4	nR VNTR 48 bp repetition	4/4	33 (66%)	31 (62%)	ns	ns	ns
		4/7	16 (32%)	18 (36%)			
		7/7	1 (2%)	1 (2%)			
	rs747302	C/C	39 (78%)	42 (84%)	ns	ns	ns
		C/G	11 (22%)	8 (16%)			
		G/G	0 (26.4%)	0 (0%)			

Notes: (a), by χ^2^-test for trend; (b), by Fisher Exact Test. ns: not significant.

**Table 3:** Dopamine receptor *(DR)* gene variants analyzed in the study.

Receptor	Gene	Variant	Change	Frequency	Effects	Score
D_1_-like						
D_1_	*DRD1*	rs4532	−48A>G	60 (%)	Association with nicotine dependence [14], tobacco smoking in schizophrenia [15], and alcohol dependence [16] and resistance to schizophrenia treatment [17].	+1
		rs686	62C>T	55 (%)	Higher *DRD1* gene expression and association with nicotine dependence [14], alcohol dependence [17], and tobacco smoking in schizophrenia [15].	+1
D_5_	*DRD5*	rs6283	978T>C	30 (%)	na	na
D_2_-like						
D_2_	*DRD2*	rs1800497	2137G>A (Taq1A)	15 (%)	Lower striatal DR D_2_ density in healthy [18].	+1
		rs6277	957C>T	50 (%)	Decreased DR D_2_ mRNA stability and translation, and reduced dopamine-induced up-regulation of DR D_2_ membrane expression in vitro [19], and lower DR D_2_ expression in cortex and thalamus of healthy subjects [20].	+1
D_3_	*DRD3*	rs6280	25G>A (Ser9Gly)	60 (%)	Higher dopamine binding affinity in vitro [21], association with alcohol dependence [22] and heroin dependence [23].	−1
		rs1800828	−712G>C	20 (%)	na	na
D_4_	*DRD4*	rs747302	−616C>G	10 (%)	No effect on DR D_4_ mRNA expression in human post-mortem brain tissue samples [23], and no association with heroin dependence [24].	na
			7 48-base pair VNTR	20 (%)	Trend toward reduced DR D_4_ mRNA expression in human post-mortem brain tissue samples [25], lower response to stimulants and requirements of higher doses of methylphenidate [26].	+1

Note: na: not applicable.

## Supplementary Materials

Supplementary materials can be found at www.mdpi.com/1422-0067/18/2/242/s1.
