# Supplementary material for: Polymorphisms of Dopamine Receptor Genes and Risk of L-Dopa–Induced Dyskinesia in Parkinson’s Disease
Source: Int J Mol Sci. 2017 Jan 24;18(2):242. doi: 10.3390/ijms18020242 (PMC5343779; doi:10.3390/ijms18020242)
Supplement: Supplementary file 1 [file ijms-18-00242-s001.pdf]

Supplementary Materials: Polymorphisms of Dopamine Receptor Genes and Risk of L-Dopa–Induced Dyskinesia in Parkinson's Disease

Cristoforo Comi, Marco Ferrari, Franca Marino, Luca Magistrelli, Roberto Cantello, Giulio Riboldazzi, Maria Laura Ester Bianchi, Giorgio Bono and Marco Cosentino

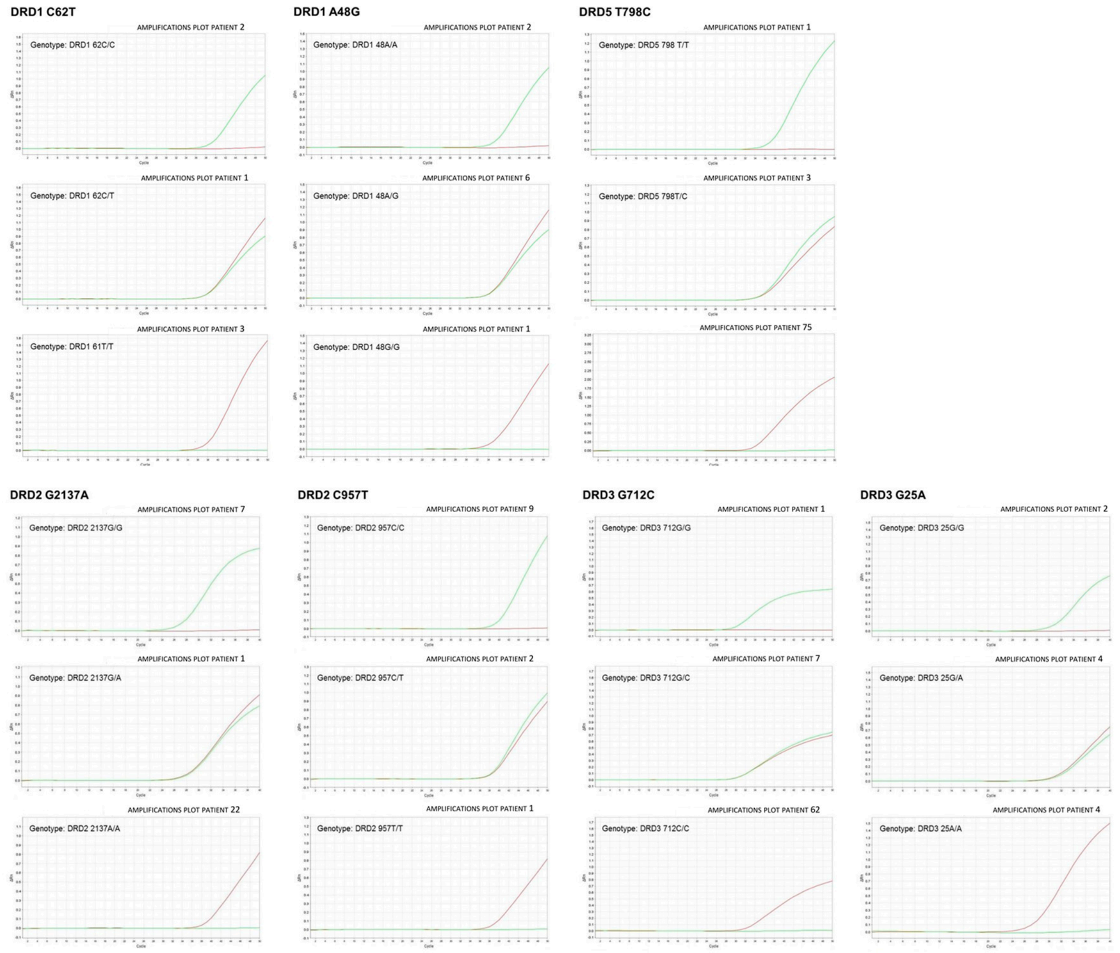

Figure S1. Example of PCR curve for each single nucleotide polymorphisms (SNP) considered in the study. Green line indicates the fluorescence associated with wild-type signal, red line indicate fluorescence associates with SNP signal.
